# Assessment of the Factors That Predict Post-Transplant Mortality in Autoimmune Liver Disease Recipients

**DOI:** 10.3390/medicina62091650

**Published:** 2026-08-28

**Authors:** Sencan Acar, Yildiray Yuzer, Yaman Tokat

**Affiliations:** 1 Department of Gastroenterology, Istanbul Florence Nightingale Hospital, Istanbul 34381, Türkiye; 2 Department of Surgery, Gayrettepe Florence Nightingale Hospital, Istanbul 34349, Türkiye; yildiray.yuzer@florence.com.tr; 3 Department of Surgery, Acibadem Hospital Group, Istanbul 34758, Türkiye; yaman.tokat@gmail.com

**Keywords:** autoimmune hepatitis, liver transplantation, primary biliary cholangitis, primary sclerosing cholangitis

## Abstract

*Background and Objectives*: Autoimmune liver diseases (AILD), including autoimmune hepatitis (AIH), primary biliary cholangitis (PBC), and primary sclerosing cholangitis (PSC), frequently lead to end-stage liver disease requiring transplantation. The factors associated with post-transplant mortality in recipients with autoimmune liver diseases (AILDs) remain incompletely characterized. We aimed to evaluate the clinical, laboratory, disease-related, and comorbidity factors associated with post-transplant mortality in this population. *Materials and Methods*: This retrospective cohort study included 94 AILD recipients (26 AIH, 28 PBC, 40 PSC) transplanted between 2004 and 2025. We analyzed clinical, laboratory, transplant-related variables, and comorbidities (Charlson Comorbidity Index [CCI]). We assessed survival using Kaplan–Meier and Cox regression analyses. Early mortality was defined as death within 90 days after transplantation. *Results*: Overall mortality was 17.0% (16/94). Mortality rates were 28.6%, 15.4%, and 10.0% in PBC, AIH, and PSC, respectively, without a statistically significant between-group difference (*p* = 0.313). Among 93 recipients with ascertainable follow-up, 8 deaths occurred within 90 days (8.6%). In the exploratory Cox regression analysis, preoperative creatinine showed the strongest association with mortality (HR = 8.67 per 1 mg/dL increase, *p* < 0.001). Transplant age (HR = 1.06 per year, *p* = 0.010) and disease duration (HR = 1.09 per year, *p* = 0.004) were also associated with mortality. These associations remained statistically significant in the exploratory multivariable model. MELD, MELD-Na, and MELD 3.0 were not significantly associated with post-transplant mortality in this cohort. Because of the limited number of deaths, the multivariable findings should be regarded as hypothesis-generating. Documented and treated acute rejection was recorded in 32.2% of recipients. In an exploratory analysis, higher BMI was associated with increased odds of this outcome; however, histopathological confirmation was not systematically available. *Conclusions*: In this single-center AILD cohort, preoperative serum creatinine, disease duration, age at transplantation, and comorbidity burden showed associations with post-transplant mortality, whereas the evaluated liver severity scores were not significantly associated with mortality. These exploratory findings require confirmation in larger multicenter cohorts before they can be incorporated into post-transplant risk-stratification models.

## 1. Introduction

Autoimmune liver disease (AILD), which includes autoimmune hepatitis (AIH), primary biliary cholangitis (PBC), and primary sclerosing cholangitis (PSC), refers to diverse conditions caused by chronic inflammatory processes resulting in progressive liver damage culminating in the development of end-stage liver disease that requires liver transplantation. Despite developments in immunosuppression therapy, AILD continues to be an important reason for liver transplantation, with a considerable number of liver transplants performed each year because of this cause.

New epidemiological information has provided a different perspective on the burden of AILD, illustrating the fact that it is both constant and changing. It has been found through analysis across the country that AILD is linked to an increased number of systemic comorbidities, such as diabetes and cancer, as well as higher mortality rates than those of the overall population, especially when PSC is present [1].

Liver transplantation remains the gold standard of treatment in cases of advanced-stage AILDs with good prospects both for immediate and future survival. However, post-transplant therapy can be difficult because of high risks of disease recurrence and immune-mediated adverse events. Disease recurrence, occurring at rates ranging from 10% to 60% depending on the disease subtype, leads to severe impairment of graft and patient survival [2]. Recurrence is primarily associated with immune system activation rather than transplantation itself.

According to recent studies, transplantation success depends not only on post-transplant biological factors but also on accessibility to the process. Intention-to-treat studies found that those with access to liver transplantation from live donors show a significantly improved overall survival rate because of less mortality during the waiting period, rather than post-transplant differences [3]. On the other hand, registry-based studies have shown mixed results across patient and donor categories, and even by disease subtype, in PSC patients [4].

However, despite these advances, one of the main limitations that still exists is that there remains an inability to understand the parameters that affect clinical outcomes of AILDs after transplantation. Most previous studies have focused on individual aspects of the disease, the immune system, or factors associated with transplantation. However, current research suggests that other factors may affect outcomes.

The current study focuses on identifying the predictors of survival in patients undergoing liver transplant surgery for autoimmune liver disease. Current research pays special attention to non-liver-specific variables and conditions that influence patient outcomes. Combining information on patients’ clinical characteristics with follow-up studies may help us develop better risk assessment methods. We additionally examined disease recurrence, acute rejection, and longitudinal post-transplant immunosuppressive patterns as secondary outcomes.

## 2. Material and Methods

### 2.1. Study Design and Population

This retrospective observational cohort study examined liver transplant recipients with AILD between 2004 and 2025 at the Florence Nightingale Hospital Group. The cohort comprised 94 patients: 26 with AIH, 28 with PBC, and 40 with PSC. Exclusion criteria included multiple organ transplants, acute liver failure, and incomplete medical records. We included variant syndromes and categorized them by phenotype. The Ethics Committee of Kosuyolu High Specialization Education and Research Hospital approved this study (reference: [2026/05/1408]); informed consent was waived.

### 2.2. Diagnostic Classification

AILD subtypes were diagnosed based on established international criteria: AIH: International Autoimmune Hepatitis Group criteria; PBC: Cholestatic liver enzyme profile with positive anti-mitochondrial antibodies (AMA) and/or consistent histopathology; PSC: Cholangiographic evidence on magnetic resonance cholangiopancreatography (MRCP) or endoscopic retrograde cholangiopancreatography (ERCP). Patients were grouped by primary diagnosis.

### 2.3. Data Collection and Variables

Pre-transplant variables included demographic information (age, sex, height, weight, body mass index [BMI]); disease-related information (diagnosis, date of diagnosis, disease duration), clinical features (varices, icterus, ascites, hepatic encephalopathy, hepatorenal syndrome, spontaneous bacterial peritonitis); comorbidities assessed using the Charlson Comorbidity Index (CCI). Laboratory parameters included liver/kidney function tests, immunological markers (ANA, AMA, LKM, ASMA, SLA), immunoglobulins (IgA, IgM, IgG), and disease severity scores (MELD, MELD-Na, MELD 3.0, Child-Pugh). Post-transplant follow-up occurred on day 7, at months 3 and 6, and at years 1, 2, 5, and 10.

### 2.4. Outcomes

The primary outcome was all-cause mortality. Secondary outcomes included recurrence of disease, documented and treated acute rejection, documented chronic rejection, re-transplantation, biliary intervention, and osteoporosis/bone fracture. Follow-up time was calculated from the date of transplantation to death or the last documented clinical follow-up.

Acute rejection was operationally defined as a documented post-transplant clinical diagnosis of acute rejection that resulted in targeted anti-rejection treatment, including corticosteroid pulse therapy, corticosteroid recycling, intensified maintenance immunosuppression, or combination therapy. The retrospective dataset recorded the first documented rejection event for each recipient rather than the total number of rejection episodes.

Histopathological confirmation was not required for inclusion in this outcome because liver biopsy reports and Banff Rejection Activity Index scores were not systematically available over the study period. Accordingly, this outcome represents documented and treated acute rejection and should not be interpreted as exclusively biopsy-proven acute T-cell–mediated rejection. Chronic rejection was assessed retrospectively through the available histopathology records and documented indications for re-transplantation. A case was classified as chronic rejection only when the diagnosis was explicitly documented in a liver biopsy report or recorded as a clinical indication for re-transplantation. Because protocol surveillance biopsies were not performed and complete longitudinal histopathological data were unavailable, chronic rejection was evaluated descriptively and was not included in regression analyses.

Post-transplant immunosuppressive treatment was administered according to the center’s prevailing protocols and individualized according to the underlying AILD subtype, rejection history, renal function, infectious complications, and treatment-related adverse effects. Longitudinal post-transplant immunosuppressive patterns were evaluated as a secondary descriptive outcome to provide clinical context for the analyses of acute rejection and disease recurrence; they were not considered pre-transplant predictors of mortality.

Detailed information on pre-transplant immunosuppressive exposure -including the specific agents used, treatment duration, cumulative corticosteroid dose, treatment response, and treatment-related adverse events- was not systematically available in the retrospective records. Therefore, pre-transplant immunosuppressive therapy could not be reliably reconstructed or included as a covariate.

### 2.5. Statistical Analysis

Statistical analyses were performed using Python 3.12 (pandas, NumPy, SciPy, lifelines, scikit-posthocs, statsmodels, matplotlib, seaborn). Confirmatory analyses were performed in Jamovi (Version 2.6.44, The Jamovi Project, 2025, https://www.jamovi.org (accessed on 4 July 2026)) and JASP (Version 0.95.4.0, Jeffreys’s Amazing Statistics Program, 2025, https://jasp-stats.org (accessed on 4July 2026)). We estimated survival curves using the Kaplan–Meier approach and tested between-group differences using the log-rank, Breslow, and Tarone–Ware tests. We considered disease groups in a three-group analysis (AIH vs. PBC vs. PSC) and a two-group analysis (hepatocellular [AIH] vs. cholestatic [PBC + PSC]). We investigated potential predictive factors using univariable Cox proportional hazards regression with demographic variables, donor variables, pre-surgery laboratory values, prognostic scores (MELD, MELD-Na, MELD 3.0, Child-Pugh, CCI), decompensation factors, recurrence of disease, and acute rejection. We verified the proportional hazards assumption using Schoenfeld residuals. Given the limited number of deaths, we considered the multivariable Cox analysis exploratory and did not intend to develop or validate a clinical prediction model. We restricted model complexity to three clinically relevant, non-collinear covariates. We assessed multicollinearity using Spearman’s rank correlation coefficients (ρ ≥ 0.80). Discrimination was assessed by Harrell’s c-index. We performed prespecified subgroup analyses for the cholestatic group (PBC + PSC). We estimated the dynamics of postoperative creatinine concentration using linear mixed-effects models with random intercepts. We tested the time-by-group interaction at five postoperative time points (months 3 and 6, and years 1, 2, and 5). Early post-transplant mortality was defined as death occurring within 90 days after liver transplantation, whereas deaths occurring beyond 90 days were classified as late mortality. Thirty-day mortality was additionally reported descriptively. We excluded one patient without an ascertainable follow-up date from the time-dependent mortality analyses. Given the limited number of early deaths, comparisons between patients with and without early mortality were restricted to exploratory univariable analyses. Continuous variables were compared using the Mann–Whitney U test, and categorical variables were compared using Fisher’s exact test, as appropriate. We did not construct a multivariable model for early mortality because of the limited number of events and the associated risk of overfitting. Two-sided *p*-values ≤ 0.05 were considered statistically significant.

## 3. Results

### 3.1. Patient Characteristics and Intergroup Differences

A total of 94 recipients with AILD underwent liver transplantation: 26 with AIH, 28 with PBC, and 40 with PSC. Table 1 summarizes baseline recipient demographic, anthropometric, disease-related, and transplant characteristics. Age at transplantation differed significantly (*p* = 0.010); the PBC group had a higher average transplant age (51.4 ± 11.2 years), whereas the PSC group underwent transplantation at a younger age (40.6 ± 15.0 years). The difference between the PBC and PSC groups was statistically significant; meanwhile, the AIH group did not differ from the other groups. Disease duration was similar across all groups, with a median of 4.9–5.3 years (*p* = 0.941). The sex ratio differed significantly (*p* < 0.001): all patients with AIH and 92.9% of PBC patients were female, while the PSC group was predominantly male (62.5%). The body mass index was significantly higher in the AIH group than in the PSC group (*p* = 0.005). No statistically significant differences were observed in the distribution of blood groups and Rh factors between the recipient groups. The presence of a variant syndrome (four PSC + AIH, six PBC + AIH, two AIH + PBC) and accompanying autoimmune diseases (12 ulcerative colitis [UC], one Crohn’s disease, two celiac disease, one systemic lupus erythematosus [SLE], one UC plus celiac disease, three thyroiditis, one psoriasis, and one type 1 diabetes mellitus) was found in 36.2% of patients, which was not statistically significant among groups (*p* = 0.125); however, the prevalence of subtypes was different (*p* < 0.001). In the PSC group, UC accounted for 78.9% of cases, whereas the AIH variant accounted for 85.7% in the PBC group.

### 3.2. Transplant and Surgical Features

Living donor LT was the most common type of transplant procedure in all groups; meanwhile, the use of a cadaveric donor was statistically significantly more common in the PBC group (28.6%; *p* = 0.003). The technique of biliary anastomosis also differed significantly (*p* < 0.001); duct-to-duct anastomosis was the most common in AIH (92.0%) and PBC (85.2%) groups, while Roux-en-Y anastomosis was applied in 51.3% of PSC patients (Table 1).

### 3.3. Preoperative Clinical and Laboratory Findings

Table 2 presents pre-transplant hepatic decompensation findings, prognostic scores, laboratory parameters, and available autoantibody results. In relation to preoperative hepatic decompensation manifestations, ascites occurred significantly more frequently in AIH patients (73.1%) and PBC patients (81.5%) than in PSC patients (42.1%; *p* = 0.002). Similar findings were observed for hepatic encephalopathy, which was noted in 42.3% of AIH patients, 38.5% of PBC patients, and only 5.4% of PSC patients (*p* = 0.001). The occurrence of esophageal varices, jaundice, variceal bleeding, and spontaneous bacterial peritonitis did not differ significantly between the groups. In terms of prognostic scores, the Child-Pugh, MELD, MELD-Na, MELD 3.0, and Charlson Comorbidity Index (CCI) scores were evenly distributed across groups (*p* > 0.05). Regarding preoperative laboratory findings, cholestatic enzymes showed significant intergroup differences, with GGT and ALP levels significantly higher in PSC patients than in those with AIH or PBC (both *p* < 0.001). Aminotransferase levels also differed between groups, with AST and ALT significantly higher in PSC (*p* = 0.016 and *p* = 0.001, respectively). The INR was significantly higher in AIH than in PSC patients (*p* = 0.019). No difference was detected with respect to total bilirubin, albumin, creatinine, and sodium levels. In the preoperative autoantibody test profile, AMA positivity was significantly higher in PBC patients (83.3%) than in AIH patients (25.0%) and PSC patients (7.1%; *p* < 0.001), whereas ANA positivity was statistically similar between the three groups (Table 2).

### 3.4. Donor Characteristics

Donor characteristics did not vary markedly between groups. Donor age, sex, body mass index, blood group, Rh factor, and degree of kinship did not differ significantly (all *p* > 0.05). Two-thirds of the donors were first-degree relatives.

### 3.5. Postoperative Clinical Outcomes

Table 3 summarizes post-transplant mortality, disease recurrence, acute rejection, re-transplantation, biliary interventions, and bone-related outcomes. Overall, the postoperative mortality rate was 17.0% (16 of 94 patients). Mortality rates were 28.6%, 15.4%, and 10.0% in PBC, AIH, and PSC patients, respectively. No statistically significant differences were found among the three groups (*p* = 0.313). Cholangiosepsis accounted for the majority of deaths (6 out of 16, 37.5%). Graft failure (3 out of 16, 18.8%), perioperative bleeding (2 out of 16, 12.5%), and other causes (5 out of 16, 31.2%) were noted among deaths. Median follow-up time was similar across groups (39.4 to 46.1 months; *p* = 0.661). Disease recurrence was seen in 13 cases (14.8%) with available data. Recurrence rates and time to recurrence did not differ significantly between groups. Acute rejection was observed in 29 of 90 recipients with available data (32.2%), with no significant differences between groups (*p* = 0.452). The median time to acute rejection was less than one month in each disease group (0.4–0.6 months). Treatment approaches to acute rejection included pulse steroid therapy (31.0%), steroid recycling (250 mg or 100 mg initial dose, rapid-tapering regimen) (27.6%), and other combination therapy (41.4%), which was equally distributed across groups. Re-transplantation was required in 7 of 88 recipients (7.9%) and was similarly distributed among the groups (*p* = 0.999). The requirement for PTC or ERCP varied across groups (*p* = 0.046); PSC (38.9%) and AIH (33.3%) had a higher incidence of biliary interventions than PBC (11.1%). Data on osteoporosis were incomplete since only half of the sample had data available, but there was no significant difference among the groups (*p* = 0.429). Bone fractures were detected only in the AIH group (3 out of 12, 25.0%), with statistical significance (*p* = 0.013) (Table 3).

### 3.6. PTC/ERCP Requirement According to the Biliary Anastomosis Technique

We analyzed PTC or ERCP requirements by biliary anastomosis technique in 85 patients. The rates were 40.0% for Roux-en-Y anastomosis, 25.9% for duct-to-duct anastomosis, and 28.6% for duct-to-sheet anastomosis. These results did not differ significantly (*p* = 0.468).

### 3.7. Immunosuppressive Therapy

Longitudinal post-transplant immunosuppressive patterns are summarized below as a secondary descriptive analysis intended to provide clinical context for the rejection and recurrence findings. Utilization of immunosuppressive drugs was evaluated at seven postoperative time points. The use of steroids was 100% on day 7 and at month 3, followed by a progressive decrease to 50.0–71.4% among patients with available data at year 10. A consistent between-group difference in mycophenolate mofetil (MMF) use was observed, with MMF use consistently higher in the AIH group than in the other two groups. The differences were statistically significant at month 6 (*p* = 0.008), year 1 (*p* = 0.019), and year 2 (*p* = 0.002), consistently reflecting differences between the AIH–primary cholestatic spectrum groups. Tacrolimus use remained high across all groups at all time points, ranging from 71.4% to 93.8%, with no significant between-group differences. Cyclosporine use was relatively uncommon (4.0–28.6%) at all time points. Tacrolimus trough levels differed between groups only on postoperative day 7 (*p* = 0.013), with higher levels in PSC than in PBC; no subsequent between-group differences were observed in tacrolimus levels or corticosteroid doses. Everolimus use was too infrequent for meaningful comparison (≤3 patients). These findings were interpreted descriptively and were not used to infer a causal relationship between specific post-transplant regimens and mortality, rejection, or disease recurrence.

### 3.8. Survival

**Figure 1:**
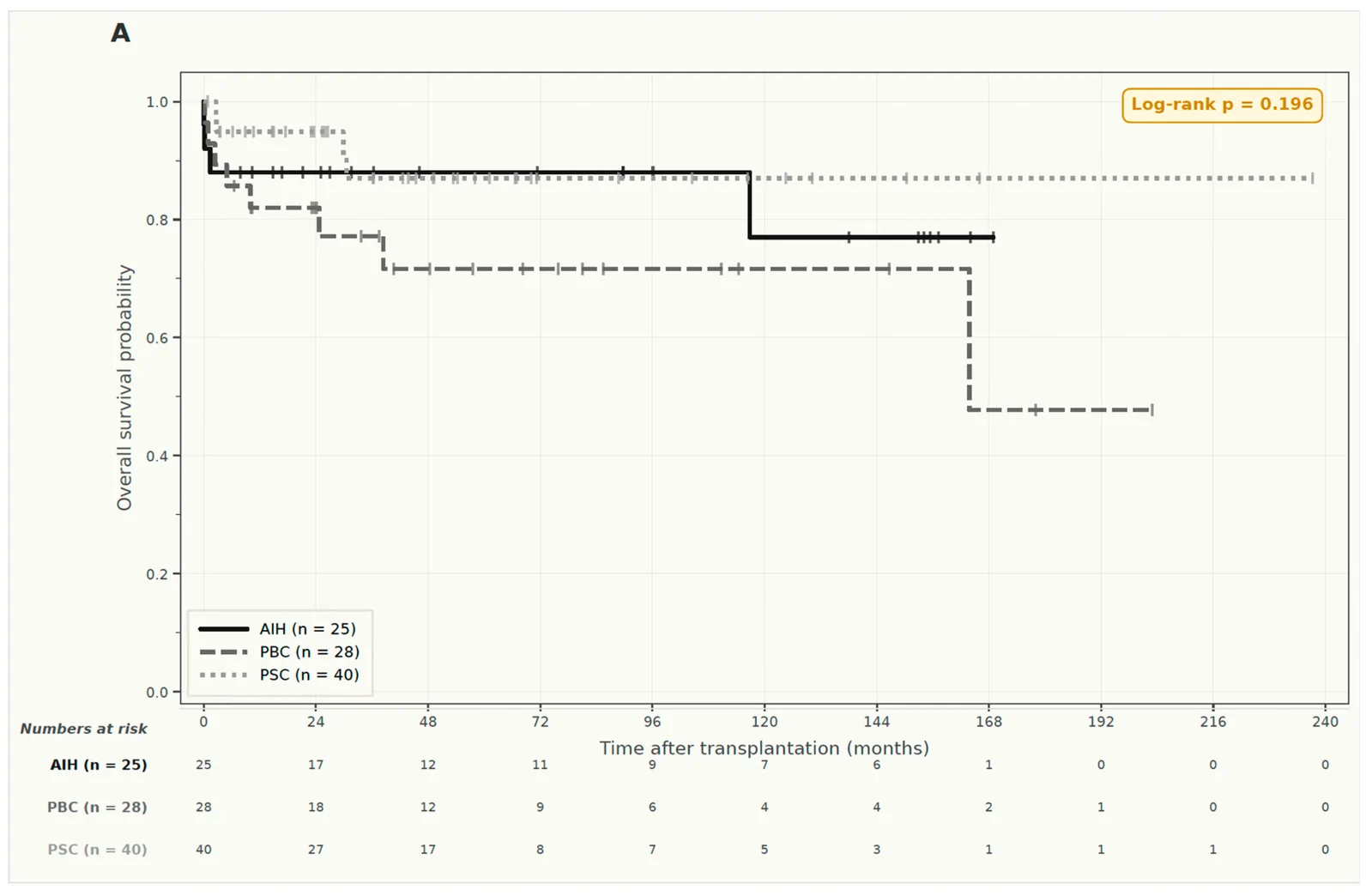

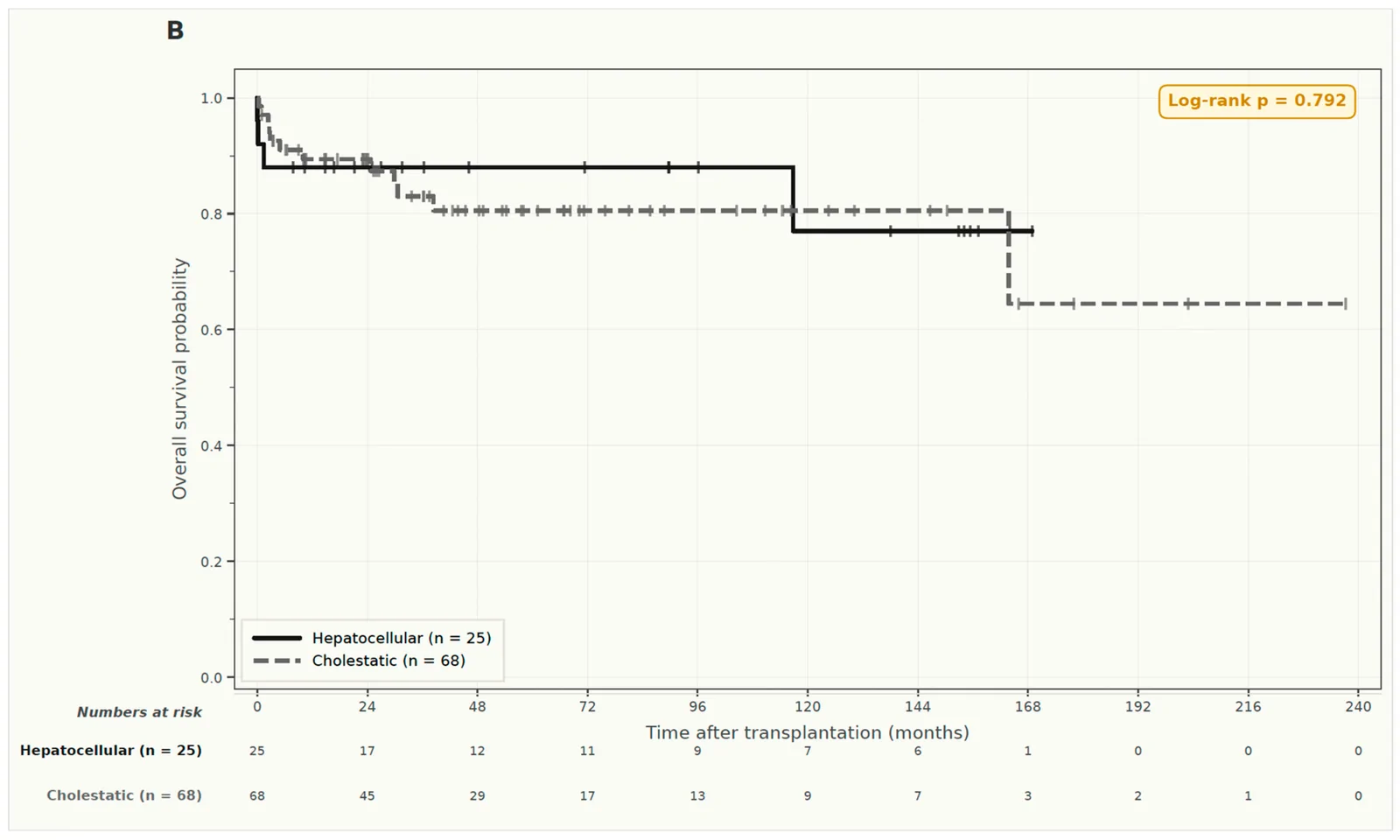
Kaplan–Meier overall survival curves. (**A**) Three-group comparison (AIH vs. PBC vs. PSC; log-rank *p* = 0.196). (**B**) Two-group comparison (hepatocellular vs. cholestatic; log-rank *p* = 0.792). Numbers at risk are shown below each panel.

Table 4 and Figure 1 present Kaplan–Meier estimates and between-group comparisons. One AIH recipient was excluded because of missing follow-up information, leaving 93 patients for survival analysis. The 1-year survival rates were 88.0% for AIH, 82.0% for PBC, and 94.9% for PSC. 5-year survival rates were 88.0%, 71.7%, and 87.0%, respectively. At 10 years, the lowest survival rate was observed in PBC (71.7%) compared with AIH (77.0%) and PSC (87.0%). Median survival was achieved only in the PBC group (163.8 months; 95% CI: 38.4–∞), whereas it was not achieved in the AIH and PSC groups. The overall difference in survival among the three groups was not statistically significant (*p* = 0.196) by the log-rank test (χ^2^ = 3.254; df = 2); results from the Breslow (*p* = 0.250) and Tarone-Ware (*p* = 0.230) tests confirmed this conclusion. Comparison of two groups (hepatocellular: AIH, n = 25; cholestatic: PBC + PSC, n = 68) showed comparable survival curves; 5-year survival rates were 88.0% and 80.6%, respectively, and no significant difference was found (log-rank *p* = 0.792; Breslow *p* = 0.957; Tarone-Ware *p* = 0.870) (Table 4) (Figure 1).

### 3.9. Factors Associated with Overall Mortality

Table 5 presents results from univariable and exploratory multivariable Cox regression analyses. In univariable analysis, age at transplantation, disease duration, CCI, and preoperative serum creatinine were significantly associated with all-cause mortality. Each additional year of age at transplantation was associated with a 6% increase in mortality hazard (HR = 1.06; 95% CI: 1.01–1.10; *p* = 0.010), while each additional year of disease duration was associated with a 9% increase (HR = 1.09; 95% CI: 1.03–1.16; *p* = 0.004). Each one-point increase in CCI was associated with an 85% increase in mortality hazard (HR = 1.85; 95% CI: 1.25–2.75; *p* = 0.002). The strongest association was with preoperative creatinine, with an 8.67-fold increase in mortality risk per 1 mg/dL (HR = 8.67; 95% CI: 2.99–25.17; *p* < 0.001). None of the other covariates, including sex, BMI, donor parameters, biliary anastomosis procedure, presence of decompensation, MELD scores, aminotransferase levels, bilirubin, albumin, and postoperative complications (recurrence and acute rejection), had a statistically significant impact on mortality in the univariate model. The proportionality assumption was violated for ascites, Child-Pugh score, MELD score, MELD-Na score, MELD 3.0 score, ALP, and INR; therefore, these results should be interpreted with caution. Given the low number of events (n = 16), we developed an exploratory multivariable model. Age at transplantation (adjusted HR = 1.05; 95% CI: 1.01–1.10; *p* = 0.029), disease duration (adjusted HR = 1.11; 95% CI: 1.04–1.18; *p* = 0.002), and preoperative creatinine (adjusted HR = 8.08; 95% CI: 2.53–25.78; *p* < 0.001) remained significantly associated with mortality (Harrell’s C = 0.780; likelihood ratio χ^2^ = 25.58; *p* < 0.001). We did not include CCI and transplant age in the same model because of high collinearity (Spearman ρ = 0.85). Given the relatively low events-per-variable (EPV) ratio (5.3), interpret the multivariate results with caution (Table 5).

### 3.10. Early Versus Late Post-Transplant Mortality

Among the 93 patients with ascertainable follow-up, four deaths occurred within 30 days after transplantation, corresponding to a 30-day mortality rate of 4.3%. Eight deaths occurred within 90 days, yielding an early mortality rate of 8.6%, whereas the remaining eight deaths occurred beyond 90 days.

Early deaths were observed in three patients with AIH, three with PBC, and two with PSC, with no significant difference according to disease group. The causes of early mortality were cholangiosepsis in three patients, perioperative bleeding in two, graft failure in one, septic shock with acute respiratory distress syndrome in one, and small-for-size syndrome in one.

In exploratory univariable comparisons, patients who experienced early mortality were older at transplantation than those without early mortality (median, 60.7 vs. 46.0 years; *p* = 0.009), had a higher Charlson Comorbidity Index (median, 4.5 vs. 3.0; *p* = 0.010), and had higher preoperative serum creatinine levels (median, 1.0 vs. 0.6 mg/dL; *p* = 0.042). MELD, MELD-Na, and MELD 3.0 scores were numerically higher among patients with early mortality but did not reach statistical significance. Because only eight early deaths occurred, we did not construct a multivariable model.

#### Documented and Treated Acute Rejection

At least one documented and treated acute rejection event was recorded in 29 of 90 recipients with available data (32.2%). The proportions were 41.7% in AIH, 32.1% in PBC, and 26.3% in PSC recipients, without a statistically significant between-group difference (*p* = 0.452). The dataset captured the first documented event per recipient and did not permit reliable determination of the total number of rejection episodes. Histopathological information was not systematically available. Only two available pathology records explicitly described acute cellular rejection, and Banff Rejection Activity Index scores could not be retrieved. We identified the remaining events from documented clinical diagnoses and the initiation or intensification of anti-rejection treatment. Therefore, the reported outcome should not be interpreted as the incidence of biopsy-proven acute T-cell–mediated rejection alone.

Exploratory Firth-penalized logistic regression results are presented in Table 6. Univariate analysis revealed that only BMI was statistically significant (OR = 1.11; 95% CI: 1.01–1.23; *p* = 0.026); each 1-unit increase in BMI was associated with an 11% increase in the odds of rejection. Gender approached statistical significance (*p* = 0.061), whereas disease duration (*p* = 0.167) and MELD score (*p* = 0.123) met the inclusion criterion for the multivariate model (*p* < 0.200). In the exploratory multivariable model (n = 85; 29 events; EPV = 7.2), BMI remained associated with documented and treated acute rejection (adjusted OR = 1.11; 95% CI: 1.00–1.22; *p* = 0.045). None of the variables, including disease group, transplant age, variant syndrome, donor type, Child-Pugh score, and CCI, were statistically significant predictors of acute rejection. The model had moderate discriminative capacity (AUC = 0.710; 95% CI: 0.585–0.825) (Table 6). Among the 29 affected recipients, 9 received corticosteroid pulse therapy, 8 received a corticosteroid recycling regimen, and 12 received other intensified or combination immunosuppressive treatments.

### 3.11. Chronic Rejection

Chronic rejection was explicitly documented in two recipients (2.1%): one with AIH and one with PBC. No documented chronic rejection was identified in the PSC group. In the AIH recipient, chronic rejection was recorded as the indication for re-transplantation, with medication non-adherence noted in the clinical record. In the PBC recipient, chronic rejection was documented histopathologically following a previous early re-transplantation performed for portal vein thrombosis; this patient subsequently died from graft failure. Because only two cases were identified, we did not perform a between-group comparison or regression analysis.

### 3.12. Factors Associated with the Need for PTC/ERCP

For factors associated with the need for PTC or ERCP, only the autoimmune disease group met the omnibus test criteria (*p* = 0.012). The odds of undergoing PTC or ERCP were numerically lower in PBC recipients than in AIH recipients, but the difference was not statistically significant (OR = 0.28; 95% CI: 0.07–1.15; *p* = 0.077). None of the following variables were significantly associated with the need for PTC or ERCP: transplant age, gender, biliary anastomosis, donor, Child-Pugh score, or MELD score. The model’s discriminative ability was poor, with an AUC of 0.649 (95% CI: 0.541–0.753).

### 3.13. Postoperative Laboratory Variables

We analyzed postoperative laboratory measures using cross-sectional analyses and linear mixed-effects models at five follow-up points (months 3 and 6 and years 1, 2, and 5). In the early postoperative period (month 3), AST and ALT levels were significantly higher in the AIH group than in the other groups (*p* = 0.026 and *p* = 0.009, respectively). These differences disappeared during later follow-up periods, and aminotransferase levels became equal between groups starting from month 6. In year 2, serum bilirubin levels were significantly higher in PSC patients than in PBC patients (*p* = 0.048). At year 5, AST levels also differed significantly between groups, with higher levels in AIH than in PBC (*p* = 0.011). GGT levels at year 5 were borderline statistically significant (*p* = 0.050). Creatinine, ALP, albumin, and INR levels did not differ significantly between groups at most follow-up points. The linear mixed effects model largely confirmed the cross-sectional results; the time × group interaction was statistically significant only for creatinine (*p* = 0.015). The time, group, and interaction effects for other laboratory measures were not statistically significant. ICC values ranged between 0.269 (INR) and 0.600 (GGT).

### 3.14. Recurrence

We determined factors associated with disease recurrence using Firth’s penalized logistic regression (n = 88; 13 events). Univariate analysis revealed that four variables showed statistical significance: MELD score (OR = 1.12; 95% CI: 1.01–1.23; *p* = 0.032), MELD-Na score (OR = 1.10; 95% CI: 1.00–1.21; *p* = 0.040), MELD 3.0 score (OR = 1.10; 95% CI: 1.01–1.21; *p* = 0.032), and total bilirubin (OR = 1.07; 95% CI: 1.01–1.14; *p* = 0.026). These variables represent the severity of preoperative liver disease and are mathematically dependent. Therefore, we used the MELD score as the representative variable in multivariable analysis, along with sex (based on theoretical considerations of sex-specific immune systems in autoimmune liver diseases). Other factors, including disease group, age at transplant, disease duration, donor type, bile duct reconstruction, Child-Pugh score, Charlson Comorbidity Index (CCI), albumin, and acute rejection, were not associated with recurrence risk. In the exploratory multivariable model, neither MELD score (adjusted OR = 1.10; 95% CI: 0.99–1.22; *p* = 0.067) nor male sex (adjusted OR = 0.30; 95% CI: 0.05–1.85; *p* = 0.196) reached statistical significance. However, the model was statistically significant overall (penalized LR χ^2^ = 13.80; *p* = 0.001). Given the low number of events (EPV = 6.5), these results are exploratory.

### 3.15. Comparison of Hepatocellular (AIH) vs. Cholestatic (PBC + PSC) Groups

In the analysis comparing hepatocellular (AIH, n = 26) and cholestatic (PBC + PSC, n = 68) patients, there were no statistically significant differences in age at transplantation or disease duration (*p* = 0.526 and *p* = 0.975, respectively). Significant differences were observed in sex distribution: all hepatocellular patients were women, whereas the cholestatic group included 60.3% women (*p* < 0.001). Body mass index (BMI) differed significantly, with higher values in the hepatocellular group (26.3 ± 5.8 vs. 23.4 ± 4.2; *p* = 0.009). Living donor transplantation was observed in all hepatocellular patients, compared with 83.8% of cholestatic patients (*p* = 0.031). The difference in biliary anastomosis technique was statistically significant (*p* = 0.004); Roux-en-Y anastomosis was used in the cholestatic group (31.8%). Among decompensation findings, only hepatic encephalopathy was significantly more prevalent in the hepatocellular group (42.3% vs. 17.6%; *p* = 0.013); all other decompensation findings and prognostic scores (Child-Pugh, MELD, MELD-Na, MELD 3.0, and CCI) were comparable between the groups. Clinical outcomes, including overall mortality, disease recurrence, acute rejection, re-transplantation, and PTC/ERCP requirement, did not differ significantly between the hepatocellular and cholestatic groups (all *p* > 0.05) (Supplementary Table S1).

### 3.16. Factors Associated with Mortality in the Cholestatic Group (PBC + PSC)

In the cholestatic group (PBC + PSC, n = 68; 12 events), we assessed the association between signs of decompensation before surgery and prognostic scores for overall survival using univariate Cox regression analysis. There was no association found between any of the decompensation signs (esophageal varices, jaundice, ascites, variceal bleeding, hepatic encephalopathy) and mortality (all *p* > 0.05). Among the prognostic scores, only the Charlson Comorbidity Index (CCI) was associated with mortality (hazard ratio [HR] = 1.60; 95% CI: 1.01–2.55; *p* = 0.047); a one-point increase in CCI was associated with a 60% higher risk of mortality. In contrast, Child-Pugh, MELD, MELD-Na, and MELD 3.0 scores were not significantly associated with survival in the cholestatic group. For the Child-Pugh score, there was a violation of the proportional hazards assumption (Schoenfeld *p* = 0.030); therefore, this result should be carefully interpreted (Supplementary Table S2).

### 3.17. Descriptive Data of 10-Year Survivors

We descriptively evaluated 20 patients with data available at the postoperative year 10 time point (AIH = 7, PBC = 7, PSC = 6). This is an extremely select group of patients who have survived more than 10 years after undergoing liver transplantation. Among recipients evaluated at year 10, median aminotransferase levels were within the normal range, with median values of AST 22 U/L and ALT 18 U/L. Only one patient in the PBC group had a significantly elevated bilirubin level (13.4 mg/dL); the other tests of hepatic synthetic function (albumin, INR) were normal.

### 3.18. Laboratory and Treatment Characteristics at the Time of Recurrence

The laboratory findings at the time of disease recurrence in 13 patients are presented descriptively. The medians were as follows: AST 47 U/L, ALT 58 U/L, GGT 115 U/L, and ALP 204 U/L. Serum bilirubin levels varied widely, ranging from 0.5 to 22.4 mg/dL, with a median of 1.2 mg/dL. In the PBC subgroup, bilirubin and aminotransferase levels were higher; however, we did not make between-group comparisons because of the very small subgroup size (n = 4–5).

We analyzed immunosuppressive therapy at the time of disease recurrence in 13 patients diagnosed with recurrence. Corticosteroids and tacrolimus were prescribed in 69.2% (9/13) of patients; MMF in 30.8%; and cyclosporine in 23.1%. The median corticosteroid dose was 5.0 mg. This wide range reflects both maintenance and pulse therapy regimens. The median tacrolimus level was 7.4 ng/mL. No patient was taking everolimus at the time of disease recurrence.

## 4. Discussion

This single-center retrospective cohort study evaluated factors associated with post-transplant mortality and other clinically relevant outcomes among recipients with AIH, PBC, and PSC. Overall mortality was 17.0%, and survival did not differ significantly among the disease groups. In exploratory analyses, older age at transplantation, longer disease duration, greater comorbidity burden, and higher preoperative serum creatinine were associated with mortality, whereas Child-Pugh and MELD-based scores were not significantly associated with mortality in this cohort. These findings should be considered hypothesis-generating because of the limited number of deaths.

Overall survival was broadly consistent with previously reported outcomes after liver transplantation for AILD [2]. Although PBC recipients had numerically lower survival estimates than AIH and PSC recipients, the difference was not statistically significant in our cohort. The absence of a significant subtype-related difference is consistent with some reports but differs from studies suggesting less favorable outcomes in PSC [1,5,6]. The lack of subtype differences in our cohort may be explained by a relatively small sample size, low event numbers, and a high LDLT proportion, which may mask prognostic differences through optimized patient selection and timing. The high proportion of LDLT in this cohort (82.8%) may have contributed to favorable outcomes, as earlier access to transplantation through LDLT reduces waiting-list mortality [3,4].

The separate analysis of early mortality provides additional clinical context for the overall survival findings. Half of all deaths occurred within the first 90 days after transplantation and were predominantly attributable to infectious, perioperative, or graft-related complications. Nevertheless, the pre-transplant characteristics associated with early mortality (particularly older transplant age, greater comorbidity burden, and impaired renal function) were broadly consistent with the predictors identified in the overall survival analysis. In contrast, MELD-based scores were numerically higher but were not significantly associated with early mortality. These findings suggest that physiologic reserve and extrahepatic vulnerability may influence not only long-term survival but also the ability to withstand the immediate postoperative period. However, because the early mortality analysis included only eight events and multiple exploratory comparisons, these results should be interpreted cautiously and require confirmation in larger multicenter cohorts.

In both PSC and PBC, post-transplant complications derive predominantly from infections, malignancies, or systemic inflammation rather than liver failure. Post-transplant survival correlates only modestly with pre-transplant MELD scores [2,3,4,7,8]. Even newer MELD modifications incorporating albumin and sex showed no association with outcomes in our cohort. This suggests that while MELD effectively predicts waiting-list mortality, it fails to reliably predict post-transplant survival. Once transplantation resolves liver dysfunction, systemic parameters become relevant for survival prediction [7].

Pre-transplant creatinine showed the strongest association with overall mortality in our analysis. Although serum creatinine is incorporated into MELD-based scores, its association with post-transplant mortality should not be interpreted as demonstrating that an entirely extrahepatic construct outperformed MELD. MELD was developed primarily to estimate short-term mortality among patients awaiting liver transplantation, and its components are weighted and transformed to optimize that purpose. In the post-transplant setting, renal dysfunction contributes to mortality through perioperative acute kidney injury and calcineurin inhibitor toxicity. In the present cohort, serum creatinine, considered as an individual continuous variable, was associated with post-transplant mortality, whereas the composite MELD-based scores were not. This finding may indicate that the post-transplant relevance of renal dysfunction is not fully captured by the scores’ original mathematical structure; however, we did not formally compare predictive performance. In AILD, where immune dysregulation is central, renal dysfunction may represent low physiological reserve and increased risk of cholangiosepticemia, which is the most frequent cause of mortality in our cohort [9,10,11].

Age at transplantation and disease duration were also associated with mortality. Age may reflect reduced physiological reserve, multimorbidity, or frailty. Disease duration may reflect cumulative exposure to inflammation, decompensation, infection, and extrahepatic organ dysfunction, but it is also influenced by timing of diagnosis and may not represent true biological disease duration. In the cholestatic subgroup, CCI was associated with mortality, emphasizing the potential relevance of comorbidity burden. However, CCI was strongly correlated with age, limiting separation of their independent contributions.

In the cholestatic subgroup (PBC + PSC), CCI was the only independent predictor of mortality. This finding highlights that mortality in these diseases cannot be attributed solely to liver failure. In PSC, inflammatory bowel disease, cholangitis, and malignancy risk increase systemic burden; in PBC, autoimmune comorbidities such as Sjögren’s syndrome, thyroid disease, and metabolic bone disease similarly contribute [7]. Liver-specific scoring systems fail to capture this aspect.

Disease recurrence identified in 14.8% of patients, with no intergroup differences. Preoperative markers of hepatic dysfunction (MELD, MELD-Na, MELD 3.0, total bilirubin) were significantly associated with recurrence on univariate analysis, consistent with prior reports [2]. Most recurrent patients (10/13) were transplanted before 2015, possibly reflecting evolving immunosuppressive practices. The lack of a standardized prospective recurrence protocol may have led to underdiagnosis.

At least one documented and treated acute rejection occurred in 32.2% of recipients. In an exploratory analysis, higher BMI was associated with increased odds of this outcome, potentially through altered pharmacokinetics, metabolic inflammation, or adherence [12,13]. This finding requires caution because histopathological confirmation and Banff RAI scores were not systematically available, the dataset captured only the first documented event, and diagnostic practice may have changed over time. Outcome misclassification cannot therefore be excluded. Chronic rejection was explicitly documented in only two recipients. Although this low observed frequency is consistent with chronic rejection being an uncommon outcome in contemporary liver transplantation, underascertainment is possible because protocol surveillance biopsies were not performed and longitudinal histopathology reports were not systematically available. The small number of cases precluded evaluation of disease-specific risk factors or associations with immunosuppressive treatment.

Post-transplant immunosuppressive patterns were included to provide clinical context for the observed rejection and recurrence outcomes rather than to identify treatment-specific predictors of mortality. The higher frequency of MMF use in AIH recipients likely reflects the greater perceived need for sustained combination immunosuppression in this group [14,15]. However, treatment selection was protocol-based and clinically individualized, and may have been influenced by rejection history, renal function, infections, adverse effects, and changes in institutional practice over the long study period. Accordingly, the observed differences between disease groups should not be interpreted as evidence that a particular post-transplant regimen caused or prevented rejection, recurrence, or mortality.

Detailed pre-transplant immunosuppressive histories were not systematically available. This is particularly relevant to AIH and variant syndromes, in which prolonged corticosteroid or antimetabolite exposure may influence infection susceptibility, metabolic complications, osteoporosis, renal function, and cumulative comorbidity. Although immunosuppression is not routinely used for the underlying cholangiopathy in isolated PBC or PSC, some patients may have received it for AIH overlap, inflammatory bowel disease, or other autoimmune comorbidities. Residual confounding related to pre-transplant treatment exposure cannot therefore be excluded.

Rather than supporting immediate changes in clinical practice, these findings identify candidate variables for further investigation. Renal function, age, disease duration, and comorbidity burden may provide information not fully represented by conventional liver severity scores. The present study was not designed to develop a clinical decision tool, and these variables should not be used to modify transplant eligibility or recipient selection without confirmation in larger, prospectively characterized cohorts.

This study has several limitations. Its retrospective, single-center design limits causal inference and generalizability. Only 16 deaths occurred, restricting model complexity and increasing the risk of overfitting and unstable estimates; the reported C-index represents apparent performance and was not optimism-corrected or externally validated. The predominance of living donor transplantation may limit applicability to deceased-donor settings. Missing data affected histopathology, autoantibodies, immunoglobulins, osteoporosis, and fractures. Recurrence and rejection were not evaluated using uniform prospective surveillance protocols. Acute rejection was identified from documented clinical diagnoses and treatment, while biopsy confirmation and RAI scores were not systematically available. Chronic rejection may have been underestimated because surveillance biopsies were not performed. Detailed pre-transplant immunosuppressive exposure was unavailable. Finally, changes in surgery, supportive care, immunosuppression, and diagnostic practice over the 21-year period introduced temporal heterogeneity that could not be fully addressed.

## 5. Conclusions

In this single-center cohort of AILD recipients, preoperative serum creatinine, disease duration, and age at transplantation were associated with post-transplant mortality, while comorbidity burden was associated with mortality in the cholestatic subgroup. MELD-based and Child-Pugh scores were not significantly associated with mortality in this cohort. Given the limited number of events and the absence of model validation, these findings should be considered exploratory. Larger multicenter cohorts with standardized data collection and formal model validation are required before these variables can be incorporated into post-transplant risk-stratification tools or clinical decision-making.

## Figures and Tables

**Table 1:** Baseline Recipient Demographics, Anthropometric Measures, and Surgical Characteristics According to Primary Autoimmune Liver Disease Group.

Variables	AIH (n = 26)	PBC (n = 28)	PSC (n = 40)	*p*
**Age at transplant** (*years*)	47.1 ± 16.8 ^ab^	51.4 ± 11.2 ^a^	40.6 ± 15.0 ^b^	**0.010**
**Disease duration** (*years*) †	5.0 [0.1–26.4]	5.3 [0.1–31.0]	4.9 [0.5–16.8]	0.941
**Sex**				
Female	26 (100.0) ^a^	26 (92.9) ^a^	15 (37.5) ^b^	**<0.001**
Male	0 (0.0)	2 (7.1)	25 (62.5)	
**BMI** (*kg/m*^2^) ‡	26.4 [14.8–36.4] ^a^	23.5 [19.2–37.5] ^ab^	22.0 [14.3–31.2] ^b^	**0.005**
**Blood group**				
A	11 (42.3)	10 (35.7)	18 (45.0)	0.633
B	8 (30.8)	7 (25.0)	6 (15.0)	
AB	0 (0.0)	2 (7.1)	3 (7.5)	
O	7 (26.9)	9 (32.1)	13 (32.5)	
**Rh factor**				
Positive	24 (92.3)	24 (85.7)	36 (90.0)	0.724
Negative	2 (7.7)	4 (14.3)	4 (10.0)	
**Variant/other AI disease**, *present*	8 (30.8)	7 (25.9)	19 (48.7)	0.125
UC	0 (0.0) ^a^	1 (14.3) ^b^	15 (78.9) ^c^	**<0.001**
AIH variant	0 (0.0)	6 (85.7)	2 (10.5)	
Other	8 (100.0)	0 (0.0)	2 (10.5)	
**Donor type**				
LDLT	26 (100.0) ^a^	20 (71.4) ^b^	37 (92.5) ^ab^	**0.003**
Cadaveric	0 (0.0)	8 (28.6)	3 (7.5)	
**Biliary anastomosis type** §				
Duct-to-duct	23 (92.0) ^a^	23 (85.2) ^a^	16 (41.0) ^b^	**<0.001**
Duct-to-sheet	2 (8.0)	3 (11.1)	3 (7.7)	
Roux-en-Y	0 (0.0)	1 (3.7)	20 (51.3)	

Continuous variables are presented as mean ± SD or median [min–max]; and categorical variables are presented as n (%). Different superscripts (^a^, ^b^, ^c^) within a row indicate statistically significant pairwise differences; groups sharing the same superscript do not differ significantly. Statistical tests were selected based on distributional assumptions and expected cell frequencies. Transplant age: interval between birth and transplantation dates. Disease duration: the interval between diagnosis and transplantation. Variant syndrome percentages were calculated among patients with the syndrome (n = 34). Duct-to-duct anastomosis includes single, double, and triple anastomoses; duct-to-sheet anastomosis includes single and double anastomoses. Variables annotated with *present* indicate that only confirmed cases were included in the frequency calculations. BMI, body mass index; LDLT, living donor liver transplantation; UC, ulcerative colitis. † Data are available for 90 patients (AIH: 24, PBC: 27, PSC: 39). ‡ Data are available for 92 patients (PBC: 26). § Data are available for 91 patients (AIH: 25, PBC: 27, PSC: 39). Bold *p*-values indicate statistical significance (*p* ≤ 0.05).

**Table 2:** Preoperative Hepatic Decompensation Findings, Prognostic Scoring Systems, and Laboratory Parameters According to Primary Disease Group.

Variables	AIH (n = 26)	PBC (n = 28)	PSC (n = 40)	*p*
**Decompensation** **findings**				
Esophageal varices, present	13 (50.0)	18 (72.0)	18 (48.6)	0.151
Jaundice, present	15 (57.7)	17 (68.0)	26 (68.4)	0.636
Ascites, present	19 (73.1) ^a^	22 (81.5) ^a^	16 (42.1) ^b^	**0.002**
Variceal bleeding, present	4 (15.4)	9 (36.0)	7 (18.4)	0.155
Hepatic encephalopathy, present	11 (42.3) ^a^	10 (38.5) ^a^	2 (5.4) ^b^	**0.001**
Spontaneous bacterial peritonitis, present †	3 (11.5)	1 (4.0)	3 (8.1)	0.634
**Prognostic scores**				
Child-Pugh score	9 [5–12]	9 [6–12]	8 [5–11]	0.225
MELD score	17.5 [8–33]	17 [9–25]	15 [9–29]	0.715
MELD-Na score	19 [8–34]	18.5 [9–31]	16.5 [9–31]	0.668
MELD 3.0 score	20 [9–37]	20 [10–29]	16.5 [9–32]	0.468
CCI	3 [3–7]	4 [3–6]	3 [3–7]	0.169
**Laboratory parameters**				
Creatinine (mg/dL)	0.52 [0.20–3.00]	0.70 [0.40–1.40]	0.65 [0.35–1.30]	0.099
AST (U/L)	65 [20–763] ^ab^	60.5 [21–295] ^a^	94 [34–380] ^b^	**0.016**
ALT (U/L)	33 [8–448] ^a^	34 [11–186] ^a^	73 [15–287] ^b^	**0.001**
GGT (U/L)‡	51 [8–301] ^a^	72 [10–909] ^a^	132.5 [22–2328] ^b^	**<0.001**
ALP (U/L)	148.5 [62–331] ^a^	196.5 [53–949] ^a^	297 [122–907] ^b^	**<0.001**
Total bilirubin (mg/dL)	3.14 [0.65–35.20]	5.20 [1.30–22.50]	4.80 [1.30–39.00]	0.360
Albumin (g/dL) ‡	3.2 [1.7–5.2]	3.2 [2.3–5.2]	3.3 [2.1–4.7]	0.457
INR	1.57 [1.04–4.37] ^a^	1.40 [1.00–2.01] ^ab^	1.27 [0.93–2.42] ^b^	**0.019**
Sodium (mEq/L)	138 [126–145]	135 [120–143]	138 [118–143]	0.338
**Preoperative autoantibodies**				
ANA positive §	7/9 (77.8)	10/13 (76.9)	8/13 (61.5)	0.712
AMA positive §	3/12 (25.0) ^a^	15/18 (83.3) ^b^	1/14 (7.1) ^a^	**<0.001**

Continuous variables are presented as median [min–max] and categorical variables as n (%). Different superscripts (^a^, ^b^) within a row indicate statistically significant pairwise differences. Statistical tests were applied according to distributional assumptions and expected cell frequencies. Decompensation findings had 3.2–6.4% missing data; percentages were calculated from the available observations. Autoantibody results are reported as n/N (%) where N denotes patients with available data; ANA n = 35 (37.2%), and AMA n = 44 (46.8%). Preoperative LKM, ASMA, SLA (very low positivity, >50% missing), and IgA, IgM, and IgG (>80% missing) were excluded from the analysis. CCI, Charlson Comorbidity Index; MELD, Model for End-Stage Liver Disease; AST, aspartate aminotransferase; ALT, alanine aminotransferase; GGT, gamma-glutamyl transferase; ALP, alkaline phosphatase; INR, international normalized ratio; ANA, antinuclear antibody; AMA, antimitochondrial antibody; SBP, spontaneous bacterial peritonitis. † Fisher-Freeman-Halton exact test. ‡ Data available for 93 patients (PBC, n = 27). § Values are presented as n/N (%), where N denotes the number of patients with available data (ANA, n = 35; AMA, n = 44). Bold *p*-values indicate statistical significance (*p* ≤ 0.05).

**Table 3:** Postoperative Clinical Outcomes, Graft-Related Complications, and Biliary Morbidity According to Primary Disease Group.

Variables	AIH (n = 26)	PBC (n = 28)	PSC (n = 40)	*p*
**Overall mortality, present**	4 (15.4)	8 (28.6)	4 (10.0)	0.313
**Cause of death ‡**	*(n = 4)*	*(n = 8)*	*(n = 4)*	
Cholangiosepsis	2 (50.0)	2 (25.0)	2 (50.0)	—
Graft failure	0 (0.0)	3 (37.5)	0 (0.0)	
Perioperative bleeding	1 (25.0)	1 (12.5)	0 (0.0)	
Other	1 (25.0)	2 (25.0)	2 (50.0)	
**Follow-up duration (months)**	46.1 [0.0–168.9]	38.0 [0.3–202.9]	39.4 [0.9–237.2]	0.661
**Disease recurrence, present**	4 (18.2)	5 (18.5)	4 (10.3)	0.684
**Time to recurrence (months) §**	71.3 ± 63.2	82.4 ± 56.7	47.0 ± 21.8	0.598
Documented and treated acute rejection, ≥1 event	10 (41.7)	9 (32.1)	10 (26.3)	0.452
**Time to first documented acute rejection (months) §**	0.4 [0.1–60.2]	0.6 [0.2–5.7]	0.5 [0.1–48.2]	0.844
**Acute rejection treatment ‡**	*(n = 10)*	*(n = 9)*	*(n = 10)*	
Pulse steroid	3 (30.0)	3 (33.3)	3 (30.0)	0.999
Steroid recycle	3 (30.0)	2 (22.2)	3 (30.0)	
Other	4 (40.0)	4 (44.4)	4 (40.0)	
Documented chronic rejection	1 (3.8)	1 (3.6)	0 (0.0)	
**Re-transplantation, present**	2 (8.3)	2 (7.7)	3 (7.9)	0.999
**PTC/ERCP requirement, present**	8 (33.3)	3 (11.1)	14 (38.9)	**0.046**
PTC	4 (16.7)	2 (7.4)	6 (16.7)	
ERCP	4 (16.7)	1 (3.7)	8 (22.2)	
**Time to PTC/ERCP (months) §**	8.1 [0.5–71.9]	4.2 [0.8–5.1]	7.3 [1.5–95.7]	0.231
**Osteoporosis, present †**	7 (50.0)	3 (23.1)	6 (28.6)	0.429
**Bone fracture, present †**	3 (25.0)	0 (0.0)	0 (0.0)	**0.013**

Continuous variables are presented as mean ± SD or median [min–max]; and categorical variables are presented as n (%). Statistical tests were selected based on distributional assumptions and expected cell frequencies. Time-to-event variables were calculated from the transplantation date and are presented only among patients who experienced the event. The cause of death was observed only among deceased patients (n = 16); no statistical comparison was performed because counts were too low. Acute rejection treatment was reported among patients with rejection (n = 29): pulse steroid (methylprednisolone 500 mg × 3 days); steroid recycle (gradual dose tapering); other (MMF addition and combination regimens). Variables annotated with the present data indicated that only confirmed cases were included. Recurrence data available for 88 patients (93.6%), acute rejection in 90 (95.7%), re-transplantation in 88 (93.6%), and PTC/ERCP in 87 (92.6%). Follow-up data were available for 93 patients. PTC, percutaneous transhepatic cholangiography; ERCP, endoscopic retrograde cholangiopancreatography; MMF, mycophenolate mofetil. † Osteoporosis data were available in 48 patients (51.1%), and bone fractures in 47 patients (50.0%). ‡ Denominators differ from the total group size; percentages were calculated among patients with the relevant event (cause of death, among deceased patients, n = 16; acute rejection treatment, among patients with acute rejection, n = 29).§ Time-to-event variables were calculated from the date of transplantation and are presented only among patients who experienced the event. Bold *p*-values indicate statistical significance (*p* ≤ 0.05). The dataset recorded the presence and date of the first documented and treated acute rejection event per recipient; the total number of episodes could not be determined. Histopathological confirmation and Banff RAI scores were not systematically available. Chronic rejection was identified from explicit histopathological documentation or a documented indication for re-transplantation. No statistical comparison was performed because only two cases were identified.

**Table 4:** Kaplan–Meier Overall Survival Estimates and Between-Group Comparisons in the Total Cohort and by Hepatocellular Versus Cholestatic Disease Classification.

**Panel A.** Three-group comparison (AIH vs. PBC vs. PSC).				
**Survival estimate**	**AIH (n = 25)**	**PBC (n = 28)**	**PSC (n = 40)**	***p***
Events, n	4	8	4	—
Median survival (months) [95% CI]	Not reached	163.8 [38.4–—]	Not reached	—
1-year survival rate, % [95% CI]	88.0 [67.3–96.0]	82.0 [62.0–92.1]	94.9 [81.0–98.7]	—
3-year survival rate, % [95% CI]	88.0 [67.3–96.0]	77.2 [55.7–89.2]	87.0 [68.2–95.0]	—
5-year survival rate, % [95% CI]	88.0 [67.3–96.0]	71.7 [48.7–85.7]	87.0 [68.2–95.0]	—
10-year survival rate, % [95% CI]	77.0 [44.0–92.0]	71.7 [48.7–85.7]	87.0 [68.2–95.0]	—
**Log-rank (Mantel–Cox)**	—	—	—	**χ^2^ = 3.254, df = 2, *p* = 0.196**
**Breslow (Generalized Wilcoxon) †**	—	—	—	**χ^2^ = 2.769, df = 2, *p* = 0.250**
**Tarone-Ware †**	—	—	—	**χ^2^ = 2.936, df = 2, *p* = 0.230**
**Panel B.** Two-group comparison (Hepatocellular vs. Cholestatic).				
**Survival estimate**	**Hepatocellular (n = 25)**		**Cholestatic (n = 68)**	***p***
Events, n	4		12	—
Median survival (months) [95% CI]	Not reached		Not reached	—
1-year survival rate, % [95% CI]	88.0 [67.3–96.0]		89.4 [79.0–94.8]	—
3-year survival rate, % [95% CI]	88.0 [67.3–96.0]		83.0 [70.4–90.6]	—
5-year survival rate, % [95% CI]	88.0 [67.3–96.0]		80.6 [67.2–88.9]	—
10-year survival rate, % [95% CI]	77.0 [44.0–92.0]		80.6 [67.2–88.9]	—
**Log-rank (Mantel–Cox)**	—		—	**χ^2^ = 0.069, df = 1, *p* = 0.792**
**Breslow (Generalized Wilcoxon) †**	—		—	**χ^2^ = 0.003, df = 1, *p* = 0.957**
**Tarone-Ware †**	—		—	**χ^2^ = 0.027, df = 1, *p* = 0.870**

Kaplan–Meier method with Greenwood 95% confidence intervals. The log-rank (Mantel–Cox) test was used for primary group comparisons; Breslow (Generalized Wilcoxon) and Tarone-Ware tests were also performed. Median survival was reported as “not reached” when fewer than 50% of the patients experienced the event. One patient (ID = 72, AIH) was excluded because of missing follow-up data, and one perioperative death (ID = 75, AIH) was assigned 0.03 months (≈1 day). Analytical cohort, n = 93. Hepatocellular: AIH (n = 25); Cholestatic: PBC (n = 28) + PSC (n = 40). CI, confidence interval. † Breslow (Generalized Wilcoxon) and Tarone–Ware tests weight events by the number at risk, giving greater weight to early follow-up than the log-rank test; they are reported as supportive analyses.

**Table 5:** Univariate and Multivariable Cox Regression Analysis of Factors Associated with Overall Survival.

Variables	Crude HR [95% CI]	*p*	Adjusted HR [95% CI]	*p*
**Demographics**				
**Transplant age (years)**	**1.06 [1.01–1.10]**	**0.010**	**1.05 [1.01–1.10]**	**0.029**
Sex, male	0.87 [0.28–2.71]	0.815	—	
BMI (kg/m^2^)	1.07 [0.97–1.19]	0.174	—	
**Disease duration (years)**	**1.09 [1.03–1.16]**	**0.004**	**1.11 [1.04–1.18]**	**0.002**
Variant syndrome, *present*	1.98 [0.68–5.72]	0.208	—	
Donor type, *cadaveric* vs. LDLT (ref)	1.33 [0.37–4.84]	0.660	—	
**Biliary anastomosis**				
Duct-to-sheet vs. duct-to-duct (ref)	0.86 [0.11–6.60]	0.887	—	
Roux-en-Y vs. duct-to-duct (ref)	0.56 [0.12–2.52]	0.449	—	
**Decompensation findings**				
Esophageal varices, *present*	1.16 [0.39–3.51]	0.788	—	
Jaundice, *present*	0.51 [0.17–1.52]	0.224	—	
Ascites, *present* ^a^	0.99 [0.33–2.95]	0.988	—	
Variceal bleeding, *present*	0.90 [0.25–3.23]	0.872	—	
Hepatic encephalopathy, *present*	2.66 [0.92–7.68]	0.072	—	
**Prognostic scores**				
Child-Pugh score ^b^	1.06 [0.80–1.40]	0.680	—	
MELD score ^c^	1.02 [0.93–1.11]	0.696	—	
MELD-Na score ^d^	1.02 [0.94–1.11]	0.584	—	
MELD 3.0 score ^e^	1.02 [0.94–1.10]	0.624	—	
**CCI**	**1.85 [1.25–2.75]**	**0.002**	—	
**Laboratory parameters**				
**Creatinine (mg/dL)**	**8.67 [2.99–25.17]**	**<0.001**	**8.08 [2.53–25.78]**	**<0.001**
AST (U/L)	1.00 [0.99–1.00]	0.600	—	
ALT (U/L)	1.00 [0.99–1.01]	0.397	—	
GGT (U/L)	1.00 [1.00–1.00]	0.814	—	
ALP (U/L) ^f^	1.00 [1.00–1.00]	0.387	—	
Total bilirubin (mg/dL)	0.98 [0.92–1.05]	0.587	—	
Albumin (g/dL)	1.23 [0.59–2.55]	0.584	—	
INR ^g^	1.16 [0.48–2.79]	0.744	—	
Sodium (mEq/L)	1.02 [0.91–1.15]	0.694	—	
**Donor characteristics**				
Donor age (years)	1.01 [0.96–1.06]	0.781	—	
Donor sex, *male*	0.57 [0.19–1.73]	0.323	—	
Donor BMI (kg/m^2^)	0.92 [0.77–1.09]	0.328	—	
**Postoperative events**				
Disease recurrence, *present*	0.45 [0.09–2.17]	0.319	—	
Acute rejection, *present*	0.13 [0.02–1.02]	0.052	—	

Cox proportional hazards regression: HR > 1 indicated an increased risk of mortality. The proportional hazards assumption was assessed using the Schoenfeld residuals test. Variables with *p* < 0.200 on univariate analysis were candidates for multivariable modeling; “—” denotes variables that were not entered. ^a^ Ascites (Schoenfeld *p* = 0.049); ^b^ Child-Pugh (*p* = 0.010); ^c^ MELD (*p* = 0.012); ^d^ MELD-Na (*p* = 0.007); ^e^ MELD 3.0 (*p* = 0.007); ^f^ ALP (*p* = 0.033); ^g^ INR (*p* = 0.016); results for these variables should be interpreted with caution. Multivariable model: n = 90, events = 15, Harrell’s C = 0.780, likelihood ratio χ^2^ = 25.58 (*p* < 0.001). CCI and transplant age were not included together because of collinearity (Spearman ρ = 0.85). EPV = 5.3; therefore, multivariable results should be interpreted with caution. HR, hazard ratio; CI, confidence interval; CCI, Charlson Comorbidity Index; MELD, Model for End-Stage Liver Disease; BMI, body mass index; LDLT, living donor liver transplantation; AST, aspartate aminotransferase; ALT, alanine aminotransferase; GGT, gamma-glutamyl transferase; ALP, alkaline phosphatase; INR, international normalized ratio. Bold *p*-values indicate statistical significance (*p* ≤ 0.05).

**Table 6:** Exploratory Firth Penalized Logistic Regression Analysis of Factors Associated with Documented and Treated Acute Rejection.

Variables	Crude OR [95% CI]	*p*	Adjusted OR [95% CI]	*p*
**Disease group**				
AIH (ref)	1		—	
PBC	0.67 [0.22–2.09]	0.492	—	
PSC	0.51 [0.17–1.50]	0.221	—	
Transplant age (years)	0.99 [0.96–1.02]	0.488	—	
Sex, male	0.33 [0.11–1.05]	0.061	0.46 [0.13–1.58]	0.216
BMI (kg/m^2^)	1.11 [1.01–1.23]	**0.026**	1.11 [1.00–1.22]	**0.045**
Disease duration (years)	0.94 [0.87–1.02]	0.167	0.93 [0.85–1.02]	0.115
Variant syndrome, present	0.72 [0.28–1.84]	0.493	—	
Donor type, cadaveric vs. LDLT (ref)	0.57 [0.12–2.70]	0.480	—	
**Biliary anastomosis type**				
Duct-to-duct (ref)	1		—	
Duct-to-sheet	3.03 [0.66–13.84]	0.153	—	
Roux-en-Y	0.53 [0.16–1.74]	0.292	—	
Child-Pugh score	0.98 [0.77–1.25]	0.863	—	
MELD score	1.06 [0.98–1.15]	0.123	1.03 [0.95–1.12]	0.425
CCI	0.91 [0.60–1.38]	0.648	—	

Firth’s penalized logistic regression; profile penalized likelihood 95% CI. Five variables met the *p* < 0.200 univariate threshold; biliary anastomosis was excluded owing to sparse cells (duct-to-sheet, n = 8) and 2 df consumption. Multivariable model: n = 85, events = 29, EPV = 7.2; −2LL = 96.6, Nagelkerke R^2^ = 0.189, Hosmer-Lemeshow *p* = 0.395, AUC = 0.710 [0.585–0.825], VIF range 1.02–1.13. OR, odds ratio; CI, confidence interval; BMI, body mass index; CCI, Charlson Comorbidity Index; MELD, Model for End-Stage Liver Disease; LDLT, living donor liver transplantation. Bold *p*-values indicate statistical significance (*p* ≤ 0.05).

## Data Availability

Data is contained within the article or Supplementary Material.

## Supplementary Materials

The following supporting information can be downloaded at: https://www.mdpi.com/article/10.3390/medicina62091650/s1, Table S1. Baseline Demographics, Clinical Characteristics, and Outcomes: Hepatocellular vs Cholestatic Groups. Table S2. Univariate Cox Regression Analysis of Preoperative Decompensation Findings and Prognostic Scores with Overall Survival in the Cholestatic Subgroup (PBC + PSC, n = 68).
