# Supplementary material for: Assessment of the Factors That Predict Post-Transplant Mortality in Autoimmune Liver Disease Recipients
Source: Medicina (Kaunas). 2026 Aug 28;62(9):1650. doi: 10.3390/medicina62091650 (PMC13609270; doi:10.3390/medicina62091650)
Supplement: Supplementary file 1 [file medicina-62-01650-s001.zip › medicina-4449456-supplementary.pdf]

Supplementary Table S1. Baseline Demographics, Clinical Characteristics, and Outcomes: Hepatocellular vs Cholestatic Groups

Variables	Hepatocellular (n=26)	Cholestatic (n=68)	p
**Transplant age (years)**	47.0 ± 16.6	44.8 ± 14.5	0.526
**Disease duration (years)** [d]	5.0 [0.1–26.4]	5.0 [0.1–31.0]	0.975
**Sex**			**<0.001**
Female	26 (100.0)	41 (60.3)	
Male	0 (0.0)	27 (39.7)	
**BMI (kg/m²)** [e]	26.3 ± 5.8	23.4 ± 4.2	**0.009**
**Variant syndrome, present**	8 (30.8)	26 (38.2)	0.500
**Donor type, LDLT**	26 (100.0)	57 (83.8)	**0.031**
**Biliary anastomosis type** [f]			**0.004**
Duct-to-duct	23 (92.0)	39 (59.1)	
Duct-to-sheet	2 (8.0)	6 (9.1)	
Roux-en-Y	0 (0.0)	21 (31.8)	
**Decompensation findings**			
**Esophageal varices, present**	13 (50.0)	36 (52.9)	0.798
**Jaundice, present**	15 (57.7)	43 (63.2)	0.621
**Ascites, present**	19 (73.1)	38 (55.9)	0.127
**Variceal bleeding, present**	4 (15.4)	16 (23.5)	0.388
**Hepatic encephalopathy, present**	11 (42.3)	12 (17.6)	**0.013**
**Prognostic scores**			
**Child-Pugh score**	8.8 ± 2.0	8.5 ± 1.8	0.424
**MELD score**	17.9 ± 6.5	16.6 ± 5.5	0.309
**MELD-Na score**	19.0 ± 6.9	18.0 ± 6.3	0.485
**MELD 3.0 score**	20.7 ± 7.3	19.0 ± 6.3	0.263
**CCI**	3.0 [3.0–7.0]	3.0 [3.0–7.0]	0.908
**Outcomes**			
**Overall mortality, present**	4 (15.4)	12 (17.6)	1.000
**Disease recurrence, present** [g]	4 (18.2)	9 (13.6)	0.729
**Acute rejection, present**	10 (38.5)	19 (27.9)	0.323
**Re-transplantation, present**	2 (7.7)	5 (7.4)	1.000
**PTC/ERCP requirement, present** [h]	8 (33.3)	18 (28.1)	0.633

Continuous variables are presented as mean ± SD or median [min–max]; and categorical variables are presented as n (%). Bold p-values indicate statistical significance ($p \leq 0.05$). Hepatocellular group: autoimmune hepatitis (AIH, n=26); Cholestatic group: primary biliary cholangitis + primary sclerosing cholangitis (PBC+PSC, n=68). An independent-samples t-test was used for normally distributed continuous variables (transplant age, BMI, Child-Pugh, MELD, MELD-Na, MELD 3.0); and the Mann-Whitney U test was used for non-normally distributed continuous variables (disease duration, CCI). Pearson's chi-square test was used for categorical variables with all expected cell frequencies ≥5; Fisher's exact test was used when any expected cell frequency was <5 (sex, donor type, overall mortality, disease recurrence, and re-transplantation); Fisher-Freeman-Halton exact test was used for the R×C table of biliary anastomosis type. Normality was assessed using the Shapiro-Wilk test with supplementary evaluation of skewness and kurtosis coefficients.

[d] Disease duration data available for 90 patients (Hepatocellular n=24, Cholestatic n=66). [e] BMI data available for 92 patients (Hepatocellular n=26, Cholestatic n=66). [f] Biliary anastomosis data available for 91 patients (Hepatocellular n=25, Cholestatic n=66); percentages calculated based on available data. Duct-to-duct anastomoses include single, double, and triple duct-to-duct anastomoses; duct-to-sheet anastomoses include

single and double duct-to-sheet anastomoses. [g] Disease recurrence data available for 88 patients (Hepatocellular n=22, Cholestatic n=66); 6 patients with missing recurrence data were excluded. [h] PTC/ERCP data available for 88 patients (Hepatocellular n=24, Cholestatic n=64).
BMI, body mass index; CCI, Charlson Comorbidity Index; MELD, Model for End-Stage Liver Disease; LDLT, living donor liver transplantation; PTC, percutaneous transhepatic cholangiography; ERCP, endoscopic retrograde cholangiopancreatography.

Supplementary Table S2. Univariate Cox Regression Analysis of Preoperative Decompensation Findings and Prognostic Scores with Overall Survival in the Cholestatic Subgroup (PBC + PSC, n=68)

Variables	Crude HR [95% CI]	p
**Decompensation findings**		
**Esophageal varices, present**	0.77 [0.24–2.41]	0.651
**Jaundice, present**	0.61 [0.19–2.02]	0.420
**Ascites, present**	0.64 [0.20–2.01]	0.443
**Variceal bleeding, present**	1.11 [0.30–4.16]	0.874
**Hepatic encephalopathy, present**	1.00 [0.22–4.64]	0.999
**Prognostic scores**		
**Child-Pugh score †**	1.09 [0.78–1.52]	0.617
**MELD score**	1.02 [0.91–1.13]	0.783
**MELD-Na score**	1.02 [0.93–1.12]	0.743
**MELD 3.0 score**	1.01 [0.92–1.11]	0.781
**CCI**	1.60 [1.01–2.55]	**0.047**

Cox proportional hazards regression; HR >1 indicates increased risk of mortality. Bold p-values indicate statistical significance (p≤0.05). This subgroup analysis includes 68 patients with cholestatic liver disease (PBC + PSC) with 12 events (deaths). Due to limited events, only univariate results are presented (EPV constraint). Proportional hazards assumption was assessed using Schoenfeld residuals test; the assumption was satisfied for all variables (p>0.05) except where noted.
† Proportional hazards assumption was violated for Child-Pugh score (Schoenfeld residuals test, p=0.030). The HR should be interpreted with caution as it represents an averaged effect over time; the hazard ratio for this variable is not constant across the follow-up period.
HR, hazard ratio; CI, confidence interval; CCI, Charlson Comorbidity Index; MELD, Model for End-Stage Liver Disease.

Detailed statistical analysis explanation

All statistical analyses were performed using Python 3.12 (pandas, NumPy, SciPy, lifelines, scikit-posthocs, statsmodels, matplotlib, seaborn), with confirmatory analyses conducted in Jamovi (Version 2.6.44, The Jamovi Project, 2023, https://www.jamovi.org) and JASP (Version 0.95.4.0, Jeffreys's Amazing Statistics Program, 2024, https://jasp-stats.org). Differences were considered statistically significant at a two-sided p-value ≤ 0.05.

The normality of continuous variables was assessed separately within each disease group (AIH, PBC, and PSC) using the Shapiro-Wilk test (all subgroups n < 50). Skewness (acceptable range: −1 to +1) and kurtosis (acceptable range: −2 to +2) coefficients were additionally computed; these three components were evaluated through a 12-point composite scoring system to determine the distributional classification of each variable (10–12 points: normal; 7–9 points: borderline, treated as parametric if n > 30; 0–6 points: non-normal). Variables that failed to meet the normality assumption in any group were analyzed using nonparametric methods across all comparisons. Homogeneity of variances was using with Levene's test for normally distributed continuous variables. Normally distributed continuous variables were expressed as mean ± standard deviation, and non-normally distributed continuous variables were expressed as median [minimum–maximum]. Categorical variables were reported as frequencies and percentages. Transplant age was calculated as the interval between the date of birth and

transplantation date, disease duration as the interval between the date of diagnosis and transplantation, and follow-up duration as the interval between the transplantation date and the date of last contact or death. The biliary anastomosis type was grouped into three categories (duct-to-duct, duct-to-sheet, and Roux-en-Y); cases involving multiple anastomosis subtypes were assigned to the corresponding main category. Among patients with variant syndrome, the subtype was classified as ulcerative colitis, AIH variant, or other based on the documented comorbid autoimmune conditions. The cause of death was categorized as cholangiosepsis, graft failure, perioperative bleeding, or other on the basis of clinical records. Because of the small number of events ($n = 16$), these data were presented descriptively without statistical comparison. Acute rejection treatment was categorized as pulse steroid (methylprednisolone 500 mg × 3 days), steroid recycle (gradual dose tapering), or other (various steroid regimens, mycophenolate mofetil addition, and combination therapies) and was reported descriptively among patients who experienced rejection.

For three-group comparisons (AIH vs PBC vs PSC), one-way ANOVA was applied to normally distributed continuous variables with homogeneous variances; when the omnibus test was significant, Tukey's HSD was used for post hoc pairwise comparisons under homogeneity of variances, and Games-Howell was used under heterogeneity. Variables that met the normality assumption but exhibited heterogeneous variances were analyzed using Welch's ANOVA. The Kruskal-Wallis H test was applied to non-normally distributed continuous variables; when significant, Bonferroni-corrected Dunn's test was used for pairwise comparisons. Post-hoc results were indicated in the main tables with superscript letters ([a], [b], [c]); groups sharing the same superscript within a row did not differ significantly. For categorical variables, expected cell frequencies were inspected; Pearson's chi-square test was applied when Cochran's rule was satisfied, and the Fisher-Freeman-Halton exact test was used, otherwise; significant results were followed by Bonferroni-corrected pairwise comparisons using the chi-square or Fisher's exact test (adjusted significance level $\alpha' = 0.017$ for three pairwise comparisons). The effect sizes were reported as η^2 (eta-squared) for three-group comparisons, Cohen's d for two-group continuous comparisons, and Cramér's V for categorical comparisons. Variables with zero variance across all patients (day 7 and month 3 steroid use, year 5 and year 10 everolimus use, postoperative LKM, ASMA, SLA, and ANCA positivity), variables with ≤ 3 users (everolimus), and variables with a missing rate exceeding 80% (IgA, IgM, and IgG) were excluded from statistical comparisons and reported descriptively in the table footnotes.

Two-group comparisons between hepatocellular (AIH) and cholestatic (PBC + PSC) disease categories were performed using the independent-samples t-test, Welch's t-test, or Mann-Whitney U test for continuous variables and the chi-square or Fisher's exact test for categorical variables, in accordance with the same normality assessment framework applied to the three-group analyses.

The association between biliary anastomosis type (duct-to-duct, duct-to-sheet, Roux-en-Y) and PTC/ERCP requirement was assessed using a 3 × 2 cross-tabulation with the Fisher-Freeman-Halton exact test owing to the low expected cell frequencies. Nine patients were excluded: three with missing anastomosis data and six with missing PTC/ERCP data.

Immunosuppressive drug usage patterns were compared across disease groups at seven postoperative time points (day 7; months 3 and 6; years 1, 2, 5, and 10). At each time point, categorical drug-use variables were compared using Pearson's chi-square test or Fisher-Freeman-Halton exact test, with expected cell frequencies, and continuous variables (steroid dose and tacrolimus trough level) were calculated only among patients receiving the respective agent and compared using the Kruskal-Wallis H test. Variables remaining constant across all time points (day 7 and month 3 steroid use = 100%; year 5 and year 10 everolimus = 0%) were excluded from statistical comparison and are noted in table footnotes. Serum cyclosporine levels were not reported due to insufficient sample sizes to enable meaningful group comparisons at all time points. Progressive attrition in available sample sizes across time points (attributable to mortality, loss to follow-up, and transplantation timing) was reported separately at each time point.

Overall survival was calculated from the date of transplantation to death or last follow-up. One patient with a missing last follow-up date was excluded from the survival analysis (analytic cohort $n = 93$); a patient who died perioperatively on the day of transplantation was assigned a survival time of 0.03 months (approximately 1 day). Survival probabilities were estimated using the Kaplan-Meier method with 95% confidence intervals derived from the Greenwood formula. Survival rates were reported at clinically meaningful time points (1, 3, 5, and 10 years) with 95% confidence intervals. Median survival was designated as not reached in groups where fewer than 50% of patients experienced the event. Group comparisons were performed using the log-rank (Mantel-Cox) test for both the three-group (AIH, PBC, PSC) and two-group (hepatocellular vs cholestatic) analyses. As survival curves crossed during the follow-up period, the Breslow (Generalized Wilcoxon) and Tarone-Ware tests were

also reported. Because all deaths (n = 16) were confirmed to be disease- or transplant-related, competing risks were not modeled because of limited events. Kaplan-Meier survival curves are presented for both comparisons with accompanying numbers-at-risk tables.

Factors associated with overall mortality were evaluated using univariate Cox proportional hazards regression, and the results were expressed as crude hazard ratios (HRs) with 95% confidence intervals. The proportional hazards assumption was verified for each variable using the Schoenfeld residuals test; variables that violated this assumption were noted in the table footnotes with corresponding p-values. Variables with $p < 0.200$ on univariate analysis were considered candidates for multivariable modeling. Despite the limited number of events (n = 16 deaths; EPV = 5.3 with three covariates), an exploratory multivariable Cox regression model was constructed using the enter method to evaluate the simultaneous contribution of transplant age, disease duration, and preoperative creatinine, based on clinical relevance and univariate significance. Collinearity among candidate predictors was assessed using Spearman's rank correlation; the Charlson Comorbidity Index and transplant age were not included together owing to high collinearity ($\rho = 0.85$). Model performance was quantified using Harrell's concordance index and the likelihood ratio test. Given the constrained EPV ratio, the multivariable results were explicitly designated as exploratory results. An additional subgroup analysis was performed in the cholestatic cohort (PBC+PSC, n = 68; 12 events) to assess the association of preoperative decompensation findings and prognostic scores with overall survival using the same univariate Cox approach, which was precluded by the limited event count.

Factors associated with acute rejection (29 events) and PTC/ERCP requirement (25 events) were analyzed using Firth's penalized logistic regression. Firth's method was selected to mitigate small-sample bias and separation issues inherent in maximum-likelihood estimation with limited event counts. Confidence intervals were calculated using the profile penalized likelihood method. Univariate analysis was performed for each candidate predictor, and variables with $p < 0.200$ were considered for multivariable modeling. The linearity of continuous predictors in the logit model was assessed using the Box-Tidwell test. The number of independent variables in the multivariable models was restricted according to the EPV ≥ 10 guideline; with the Firth correction, a relaxed threshold of EPV ≥ 7 was accepted. Multicollinearity was evaluated using the variance inflation factor (VIF). Model fit was assessed using −2 log-likelihood, Nagelkerke R^2, the Hosmer-Lemeshow goodness-of-fit test, classification accuracy, and the area under the receiver operating characteristic curve (AUC) with 95% confidence intervals derived from 2000 bootstrap replications. For the PTC/ERCP requirement, only the disease group met the relaxed threshold on univariate analysis; the adjusted estimates, therefore, equaled the crude values and were not separately reported.

Factors associated with disease recurrence (13 events in 88 patients) were also analyzed using Firth's penalized logistic regression. Among the four predictors reaching univariate significance (MELD, MELD-Na, MELD 3.0, and total bilirubin), all reflected the same construct (severity of preoperative hepatic dysfunction) and were mathematically interdependent; therefore, the MELD score was therefore selected as the representative variable for the exploratory multivariable model, and sex was additionally included on biological grounds given the established role of sex-dependent immune regulation in autoimmune liver diseases. With an EPV of 6.5 for two covariates, multivariable results were explicitly designated as exploratory.

Postoperative laboratory parameters (creatinine, AST, ALT, GGT, ALP, bilirubin, albumin, and INR) were evaluated at five postoperative time points (months 3 and 6, years 1, 2, and 5). Cross-sectional three-group comparisons at each time point were performed using the Kruskal-Wallis H test; when the omnibus test was significant, Bonferroni-corrected Dunn's post hoc test was applied. To account for within-patient correlation and the unbalanced longitudinal data structure arising from progressive attrition, linear mixed-effects models with random intercepts per patient were additionally fitted for each laboratory parameter; time (categorical), group, and a time × group interaction were specified as fixed effects. Parameters with skewed distributions (AST, ALT, GGT, ALP, and bilirubin) underwent natural logarithm transformation [$\log(1 + x)$] prior to the mixed-model analysis. The significance of the time effect, group effect, and time × group interaction was evaluated using likelihood ratio tests. Year 10 data were not subjected to hypothesis testing owing to an insufficient sample size ($n \approx 20$) and were reported descriptively only. Laboratory values at the time of disease recurrence (n = 13) and the immunosuppressive treatment regimen at recurrence were similarly presented descriptively only owing to small subgroup sizes.
